# Environmental Intervention as a Therapy for Adverse Programming by Ancestral Stress

**DOI:** 10.1038/srep37814

**Published:** 2016-11-24

**Authors:** J. Keiko McCreary, Zachary T. Erickson, YongXin Hao, Yaroslav Ilnytskyy, Igor Kovalchuk, Gerlinde A. S. Metz

**Affiliations:** 1 Canadian Centre for Behavioural Neuroscience, Department of Neuroscience, 4401 University Drive, Lethbridge, Alberta, Canada; 2 Department of Biology, University of Lethbridge, Lethbridge, AB T1K3M4 Canada

## Abstract

Ancestral stress can program stress sensitivity and health trajectories across multiple generations. While ancestral stress is uncontrollable to the filial generations, it is critical to identify therapies that overcome transgenerational programming. Here we report that prenatal stress in rats generates a transgenerationally heritable endocrine and epigenetic footprint and elevated stress sensitivity which can be alleviated by beneficial experiences in later life. Ancestral stress led to downregulated glucocorticoid receptor and prefrontal cortex neuronal densities along with precocious development of anxiety-like behaviours. Environmental enrichment (EE) during adolescence mitigated endocrine and neuronal markers of stress and improved miR-182 expression linked to brain-derived neurotrophic factor (BDNF) and neurotrophin-3 (NT-3) regulation in stressed lineages. Thus, EE may serve as a powerful intervention for adverse transgenerational programming through microRNA-mediated regulation of BDNF and NT-3 pathways. The identification of microRNAs that mediate the actions of EE highlights new therapeutic strategies for mental health conditions and psychiatric disease.

The prevalence of mental health and substance use disorders has increased by 37.6% since 1990^1^. Dysregulation of the stress response represents a major risk factor for these disorders. Through programming of the stress response, prenatal stress has been shown to increase the risk of developmental disorders and psychopathologies in later life^2^,^3^. The main mechanism of developmental programming by prenatal stress involves altered hypothalamic-pituitary-adrenal (HPA) axis function and elevated stress responsiveness in the F1 generation^4^,^5^. More recently, early life stress was shown to not only influence the F1 offspring, but also future filial generations. Transgenerational programming by stress was shown to alter affective state and sensorimotor behaviour^6^, endocrine functions^7^ and heritable changes in DNA methylation status and microRNA (miRNA) expression^8^,^9^. Moreover, cumulative multigenerational stress has recently been shown to generate new behavioural traits in a sexually dimorphic manner^10^. The understanding that stress is mainly uncontrollable and transferred to filial generations highlights the need to identify interventions that can treat the adverse effects of programming by ancestral stress.

Beneficial environmental and lifestyle changes are powerful, ecologically valid strategies to mitigate adverse programming by stress. For example, exposing rodents to an enriched environment (EE) with rich social and sensorimotor stimulation decreased anxiety-like behaviours in an elevated plus maze^11^,^12^,^13^,^14^, improved sensorimotor skills^15^,^16^ and led to larger cell proliferation and neuronal density^17^ along with increased expression of brain-derived neurotrophic factor (BDNF)^18^,^19^. In prenatally stressed F1 offspring, EE decreases anxiety-like and fear behaviours^14^, improves social behaviour^20^, restores dendritic and synaptic morphology^21^,^22^ and rescues density of glucocorticoid receptors^23^. Here we aim to determine if EE represents an effective therapy for the endocrine and behavioural consequences of epigenetically inherited manifestations of transgenerational stress in rats.

The present study was designed to (1) characterize phenotypes of transgenerational and multigenerational exposure to social isolation stress along with microRNA regulation and affective behaviour, and (2) to determine if a social housing intervention by EE can mitigate the phenotype of stress induced by ancestral stress exposure. To determine the impact of EE on transgenerational versus cumulative ancestral stress, the design used (1) a lineage of transgenerational prenatal stress in which the parental generation but not the F1-F3 generations experienced stress, and (2) a lineage of multigenerational prenatal stress, in which each the parental and the offspring generations experienced gestational stress^6^,^8^,^24^,^25^. The F3 generation is of particular relevance because it is the first generation in the maternal lineage that is not directly exposed to prenatal stress and therefore changes may be considered programmed through epigenetic inheritance^26^. The focus on miRNA signatures in this study has relevance for the discovery of heritable predictive biomarkers of altered stress response. We hypothesized that ancestral stress alters HPA axis activity and affective behaviour in relation to specific miRNA signatures of stress, which then can be alleviated by EE.

## Results

### Ancestral Stress Alters HPA Axis Activity and is Alleviated by Environmental Enrichment

#### GR Receptor density

**Figure 1:**
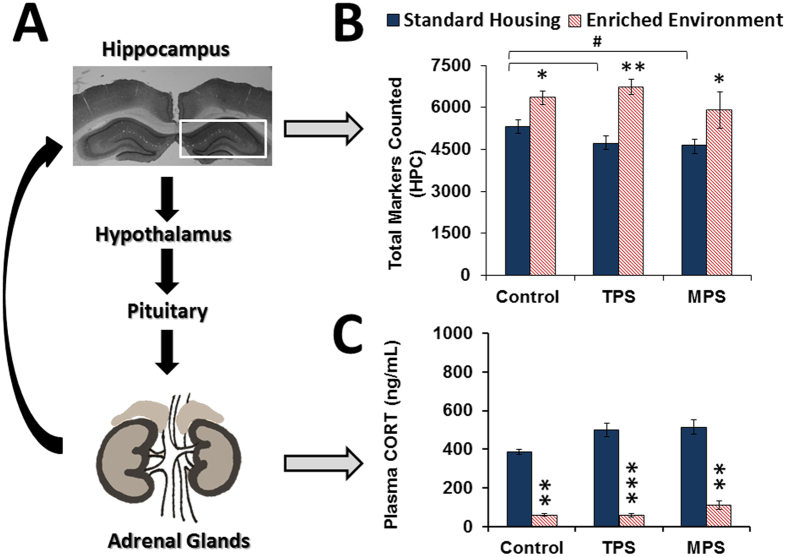
Enriched environment improves dysregulated hypothalamic-pituitary-adrenal axis activity. (**A**) Illustration of critical components of the HPA axis assessed in this study. (**B**) Both transgenerational (TPS) and multigenerational (MPS) prenatal stress reduced glucocorticoid receptor markers in the hippocampus compared to the control group. Exposure to enriched environment increased glucocorticoid receptor count. (**C**) Circulating plasma corticosterone levels were reduced by enriched environment across all groups. Enrichment therapy thus improved HPA axis feedback regulation. Asterisks denote significances due to EE (*p < 0.05, **p < 0.01, ***p < 0.001); ^#^denotes significant difference due to ancestral stress (^#^p < 0.05).

**Figure 2:**
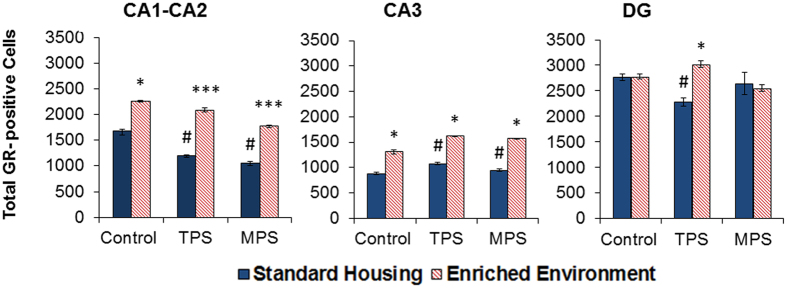
Enriched environment improves stress-programmed glucocorticoid receptor cell count. Stereological analysis of total GR-positive cells counted in hippocampal CA1-CA2, CA3 and dentate gyrus areas. Cell counts in control, transgenerationally (TPS) and multigenerationally (MPS) stressed rats revealed that ancestral stress reduced GR markers in the CA1-CA2 regions, increased markers in CA3 and decreased markers in the DG (TPS only). Enrichment increased total GR markers in all cases with the exception of the GR markers in the DG of the MPS and control animals. Asterisks denote significances due to EE (*p < 0.05, ***p < 0.001), ^#^denotes significant difference due to stress (^#^p < 0.05).

A summary of the total hippocampal GR cell count is shown in Fig. 1B and measurements of delineated areas of the hippocampus are shown in Fig. 2. Stereological analysis of total GR-positive cells (markers) counted in the hippocampus revealed a significant decrease due to stress (F(2,34) = 5.570, p < 0.05), and a significant increase due to enrichment (F(1,34) = 57.529, p < 0.0001). Delineated GR measurements included markers counted in the dentate gyrus (DG), CA1-2, and CA3. In the CA1-2 areas of the hippocampus, there was a significant main effect of stress linked to a decrease in total GR markers (F(2,34) = 15.871, p < 0.0001), and a significant effect of enriched environment linked to an increase in total GR markers (F(1,34) = 75.842, p < 0.0001). In the CA3, there was a significant increase in total GR markers due to stress compared to controls (F(2,34) = 8.423, p = 0.001), where enrichment still produced a significant increase in total markers (F(1,34) = 92.016, p < 0.0001). In the DG, there was a significant decrease in GR markers due to TPS compared to controls (p < 0.05), which was significantly improved in the enriched TPS group (p < 0.05).

#### Corticosterone

Animals that were ancestrally stressed had higher baseline CORT levels, although these results were not significant. However, enrichment significantly decreased the level of basal circulating CORT (F(1,42) = 16.16, p < 0.001) across all groups (Fig. 1C). Thus, CORT levels in enriched groups were at least on average 80% lower than in standard housing groups.

### Ancestral Stress Alters Prefrontal Cortex Neuroanatomy and Cell Density

#### Cortical Thickness

**Figure 3:**
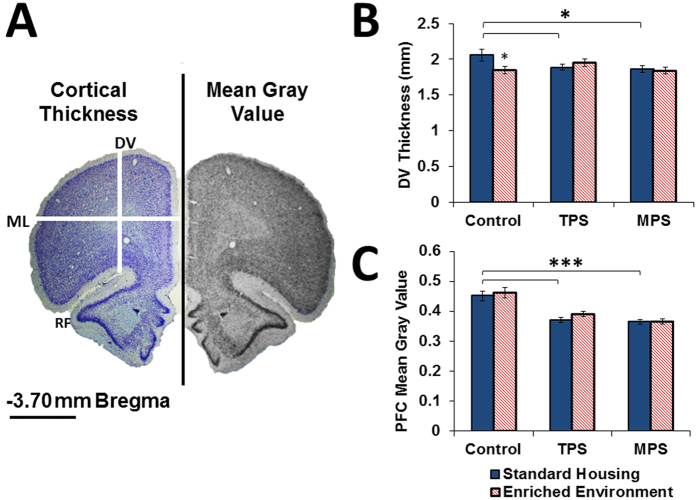
Ancestral stress reduced prefrontal cortex thickness. (**A**) Image of a representative cresyl violet-stained coronal brain section corresponding to 3.70 mm relative to bregma illustrating the measurements of cortical thickness (DV: Dorsoventral, ML: Mediolateral, RF: Rhinal fissure, scale bar represents 2 mm). The right portion shows the gray scale 8-bit image used for mean gray value analysis. (**B**) DV thickness and (**C**) mean gray value were significantly decreased due to transgenerational and multigenerational stress, with no significant effect of enrichment therapy. PFC, prefrontal cortex. Asterisks denote significances (*p < 0.05, ***p < 0.001).

Figure 3B summarizes the stress- and enrichment-associated changes in medial-lateral cortical thickness in the PFC. There was a significant effect of stress on dorsoventral thickness (F(2,103) = 8.701, p < 0.001), indicating that stressed animals had diminished cortical thickness (Fig. 3A, Left, DV). There was no significant effect of enrichment on dorsoventral cortical thickness as well as no significant effect of stress or enrichment on mediolateral thickness.

#### Mean Gray Value

A summary of stress and enrichment-associated changes in neural density in the PFC is shown in Fig. 3C. The density of the ROI drawn on the coronal sections corresponding to the caudal PFC (Fig. 3A, right) was significantly reduced in the TPS and MPS animals compared to controls (p < 0.001). There was a significant main effect of stress on mean gray values (F(2,106) = 37.456, p < 0.0001) where stressed animals had lower values, but there was no effect of enrichment.

### Ancestral Stress Leads to Precocious Hyperactivity and Risk Assessment Behaviour Which is Mitigated by Environmental Enrichment

#### Open Field

**Figure 4:**
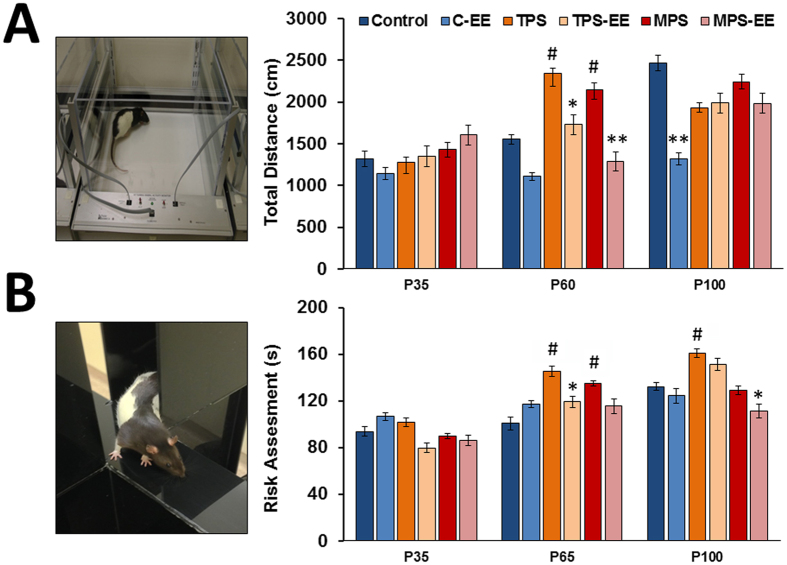
Enriched environment mitigates precocious anxiety-like behaviours induced by ancestral stress. (**A**) Photograph of a rat in the automated open field and total distance travelled measured at P35, P60 and P100 showing an increase in movement time due to ancestral stress and a decrease due to enrichment. (**B**) Photograph of a rat in stretch-extend posture in the elevated plus maze at P35, P65 and P100 observed to measure the time spent risk assessing, showing an increase in the time spent risk assessing due to ancestral stress, and a decrease due to enrichment (at P65, TPS-EE and at P100, MPS-EE). Note that transgenerational and multigenerational stress generated precocious anxiety-like behavioural traits, and enrichment therapy normalized the developmental trajectory of these behaviours. Asterisks denote significances due to EE (*p < 0.05, **p < 0.01), ^#^denotes significant difference due to stress (^#^p < 0.05).

During development (P35, P60, P100), a statistically significant main effect of Enrichment was found in all variables, indicating that enriched housing rats displayed decreased Total Distance Travelled (Fig. 4A; F(1,42) = 19.45, p < 0.001), Vertical Time (F(1,42) = 18.45, p < 0.001) and increased Margin Time (F(1,42) = 15.25, p < 0.001). The statistically significant effect of age reflects an age-related increase in distance travelled (F(2,84) = 15.28, p < 0.001) and Vertical Time (F(2,84) = 48.22, p < 0.001), and an age-related decrease in Margin Time (F(2,84) = 48.06, p < 0.001). The statistically significant interaction between Age*Environment indicates that enriched housing rats showed a greater age-related increase in Vertical Time (F(2,84) = 9.70, p < 0.001), as well as a greater age-related decrease in Margin Time (F(2,84) = 10.68, p < 0.001). Further pairwise comparisons in Total Distance Travelled indicate that stressed animals travelled significantly more at P60 (p < 0.05) compared to non-stressed animals. Pairwise comparisons also revealed that enrichment in the TPS and MPS groups showed significantly reduced distance travelled compared to the standard housing group (p < 0.05).

#### Elevated Plus Maze

During development (P35, P65, P100), the time spent in the open arms revealed a significant main effect of Age, with older rats spending more time in the open arms than younger rats (F(1.49,62.58) = 4.60, p < 0.05). Additionally, a significant main effect of Enrichment in the number of closed arm entries indicates that enriched housing rats made fewer entries into the closed arms than standard housing rats (F(1,42) = 7.12, p < 0.05). Further pairwise comparisons revealed that, at P35, enrichment resulted in an increase in time spent in open arms in MPS rats (p < 0.05); at P60 enrichment resulted in an increase in time spent in the open arms among TPS and MPS rats (p < 0.01); and, at P100, enriched TPS and MPS rats spent more time in the open arms (p < 0.01). Risk assessment behaviour also showed a significant main effect of age, (Fig. 4B; F(1,42) = 19.182, p < 0.0001) with older rats having more risk assessment behaviours. Further pairwise comparisons indicate that stressed animals had more risk assessment behaviours at P65 (p < 0.05) compared to non-stressed. Pairwise comparisons also revealed that enrichment in the TPS (P60) and MPS (P100) groups showed significantly reduced risk assessment behaviours compared to the standard housing rats (p < 0.05).

### Ancestral Stress-associated Changes in miRNA Expression are Inversely Regulated by Enriched Environment

**Figure 5:**
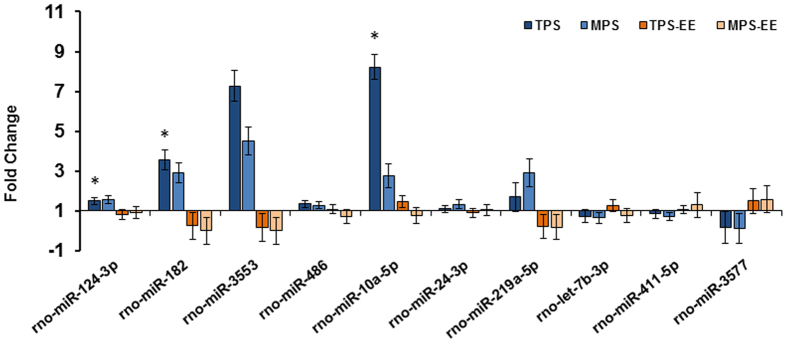
Trans- and multigenerational stress program miRNA expression profiles related to psychopathologies, which are reversed by enriched environment. Fold changes of miRNAs in TPS and MPS rats in reference to controls. Note that ancestral stress led to up- or down-regulation of miRNA expression, respectively, and enrichment returned these changes to normal levels. Asterisks denote significances (FDR adjusted; *p < 0.05).

The main small RNAs that were differentially regulated by EE and stress are summarized in Table S1.Deep sequencing revealed that 59 miRNAs (TPS: 30, MPS: 29) were differentially expressed in response to ancestral stress, and 29 miRNAs were differentially expressed in response to enriched environment (TPS-EE: 7, MPS-EE: 22). After adjusting p-values using the Benjamini and Hochberg correction^27^, three miRNAs were differently expressed which included miR-182, miR-10a-5p and miR-124-3p (FDR p < 0.05). Other miRNAs that approached significance included miR-3553, miR-24-3p, miR-219a-5p, miR-411-5p (upregulated due to stress) and miR-3577 (downregulated due to stress). Importantly, exposure to an enriched environment re-programmed the effect of ancestral stress of all miRNA changes by either upregulating or downregulating their expression, respectively. Fold changes of the miRNAs of interest differentially expressed in the animals housed in the enriched environment compared to standard housing controls are summarized in Fig. 5. Target prediction and hypergeometric test found pertinent biochemical pathways for miR-182 including neurotrophin signaling (Figure S1) and axon guidance (Figure S2). These data show that miR-182 may regulate BDNF and neurotrophin-3 (NT-3) expression and may be involved in axonal guidance mechanisms particularly involving netrin, semaphorin, and ephrin (Figures S1 and S2).

## Discussion

Ancestral exposure to a stressful environment contributes to altered stress response and may raise the risk of neuropsychiatric disease. The present study was designed to assess if a social housing intervention can mitigate transgenerational consequences of a social stressor on stress response and behaviour. Our results show that experience of stress during pregnancy in the great-grandmother leads to higher stress sensitivity, i.e., by reduced negative feedback capacity of the HPA axis, and cortical atrophy in association with an altered response to novel and aversive environments in the offspring. Ancestral stress in the F3 generation also led to precocious onset of motor hyperactivity and risk assessment behaviours in response to a novel environment. Therapeutic exposure to an environmental enrichment drastically mitigated the stress-sensitive phenotype induced by ancestral stress and improved HPA axis function across all groups. The results also provide evidence that transgenerational inheritance of altered stress response was linked to changes in miRNA expression, particularly pathways involved in brain development and affective state. EE inversely regulated the programming of stress-related miRNA signatures.

The present data demonstrate that ancestral stress modifies several levels of HPA axis function. The study used a social isolation paradigm to induce psychological stress during pregnancy as an ecologically valid analogue of human stress^28^, as opposed to the more frequently used physical stressors (e.g., restraint, shock). Female rats were isolated starting at the age of 90 days until parturition, thus withdrawing the benefit of social interaction and generating both physiological and behavioural changes. The stress severity was comparable, yet different in nature, to an established semi-random restraint and swim stress procedure used in earlier transgenerational studies^6^,^29^. TPS and MPS both reduced total hippocampal GR density, which resembles changes commonly seen after prenatal stress^4^,^30^,^31^. The changes specifically occurred in the CA1-CA2 (both TPS and MPS) and DG (TPS only) which generally show high GR density^32^. In the CA3 however, both stress and EE led to an increase in GR density. The cause for this regional difference is still unknown. We speculate, however, that because the CA3 seems to be more vulnerable to the effects of stress^33^ that there may be a protective mechanism in place for this region as a result of TPS and MPS. Nevertheless, a decrease in GR density in CA1-CA2 and DG may result in overall impaired negative feedback regulation of the HPA axis, which poses a risk for greater stress sensitivity^34^.

Negative feedback regulation is also mediated by the PFC, which was found to have reduced thickness in TPS and MPS animals. PFC neural morphology is susceptible to prenatal stress^35^, thus providing a mechanism by which ancestral stress can modulate higher-order executive functions, sensory perception and social reasoning^36^. Moreover, the PFC is part of a regulatory circuitry involved in HPA axis activity^37^. Reduced cortical thickness and diminished neural density, as induced by TPS and MPS, may therefore indicate weakened inhibitory regulation of the HPA axis^38^,^39^ and affective state^40^. Since anxiety and depressive disorders share an association with both HPA axis dysregulation and PFC function (for review see ref. 38), the current findings suggest that transgenerational programming by stress may represent a causal mechanism for such symptoms. Surprisingly, these deficits in PFC morphology were not mitigated by EE in this study, although they may have been mitigated at an earlier age that was not measured. These results agree with the notion that the PFC responds differently to EE than do regions such as the HPC^41^.

Prenatal programming of the developing brain and HPA axis has consequences that extend into adulthood, which may result in altered motor activity^2^,^4^,^6^,^42^. The present study examined animals at the age of 30, 60 and 90 days, thus covering the main stages of adolescent development. The longitudinal findings revealed that ancestral stress favors precocious onset of elevated motor activity and risk assessment behaviours in response to the novel and slightly aversive environments of open field and elevated plus maze. Both tests are standard assessments of anxiety-like behaviour and response to novelty^43^,^44^. The indication that ancestral stress enhances the vulnerability to hyperactive and anxiety-like behaviours agrees with findings made in prenatally stressed animals^45^,^46^,^47^,^48^. Our results also validate those of other studies that found precocious development of fear-related behaviours in rat pups after maternal stress^49^ and CORT exposure^50^. A possible mechanism is that stress-induced hormonal imbalance prior to pubescence may accelerate testicular development, thus increasing the brain’s exposure to androgens during development followed by a decline in testosterone during adulthood^51^. The present findings concur with notions that behavioural disorders in males become symptomatic at young ages^1^.

The formation of stress-induced precocious anxiety-related behaviour was mitigated by exposure to EE during adolescence. EE in ancestrally stressed rats reduced motor hyperactivity and anxiety-like behaviours along with lowering HPA axis activity across all groups. Reduced HPA axis activity may directly translate to reduced anxiety-like behaviour^52^. The present findings strongly suggest HPA axis activity and lifelong stress sensitivity, are programmed by ancestral stress. These consequences of adverse HPA axis programming by maternal stress can be mitigated by early beneficial experiences in the filial generations. Aside from EE exposure during adolescence, data suggest that EE at any time in life is beneficial to promote brain health and behaviour. In animals, EE promotes skilled movement^15^,^16^,^53^, learning and memory^54^,^55^ and affective behaviours^11^. In humans, enriched early childhood experiences have been associated with improved motor and cognitive function during old age^56^, lowered risk of inattentive and hyperactive/impulsive behaviours^57^, anger and fear^58^ in later life. In addition, EE was shown to decrease CORT production, further supporting the claim that EE reduces stress-associated anxiety-related behaviour^11^. It should be noted however, that the nature of EEs across studies varies considerably and results do not consistently show an improvement^59^. Yet, the present study is the first to suggest that EE, using a combination of physical and social enrichment, is capable of reducing the impact of transgenerational adverse experiences in terms of improved stress response regulation and affective state in male offspring.

The mechanisms through which ancestral stress forms new behavioural traits involves a complex interaction between genes and environment^60^,^61^. A central component of transgenerational inheritance is the transmission of epigenetic regulators across generations, including changes in DNA methylation marks^62^,^63^ and differential miRNA expression^8^,^9^. MiRNAs as post-transcriptional regulators of gene expression have particular implications as biomarkers of mental health^64^. Indeed, miRNAs that were differentially expressed in stress are recognized biomarkers of mental health, including miR-10a-5p in depression^65^, miR-219a-5p in schizophrenia^66^ and miR-182 in fear memory, depression and synaptic plasticity^67^. The present data on miR-182 suggest that an underpinning mechanism is the regulation of axonal pathfinding and synaptic maintenance during development and maturation.

Recent findings have highlighted a central role of miR-182 in structural plasticity. For example, miR-182 targets actin-regulating proteins and its upregulation was linked to disrupted amygdala-dependent fear memory^67^. Importantly, miR-182 overexpression was found to be associated with depression-like behaviours and decreased hippocampal BDNF expression in stressed rats, and miR-182 silencing led to anti-depressant-like effects^68^. Moreover, miR-182 has been shown to regulate BDNF expression^69^, thus proposing a key mechanism for stress to interact with the expression of affective behaviours. The miRNA downregulation by EE suggests that improved HPA axis regulation, through miRNA regulation and other epigenetic mechanisms may restore cortical and hippocampal BDNF levels to support neuronal maintenance and neuroplasticity during brain maturation. Indeed, EE has been shown to influence behaviour and brain plasticity mainly through the action of BDNF^19^,^70^.

Further studies have also associated miR-182 with the regulation of NT-3 expression. In particular, enhanced neuroplasticity and improved learning and problem-solving ability in EE-exposed rats was associated with higher cortical NT-3 mRNA expression^71^. However, compared to a large body of research focusing on EE-induced changes in BDNF expression, the regulation of NT-3 received less attention. NT-3 acts as a potent regulator of neuronal survival, neurogenesis, differentiation and synapse formation^72^, which may explain at least in part the therapeutic impact of EE on brain development and maturation.

Using an animal model, the present data suggest that consideration of ancestral stress is critical to the understanding of mental health manifestations in the absence of genetic determinism. Ancestral stress led to precocious developmental trajectories of anxiety-like behaviour during adolescence along with elevated HPA axis activity and altered miRNA expression. The present observations also suggest that EE may serve as a therapy to effectively mitigate generationally programmed stress sensitivity and adverse health outcomes in males, along with potentially re-programming their pathogenic epigenetic pathways. As results from this study suggest the probability of formation of depressive-like behaviours, future research in animal models of ancestral stress may consider the evaluation of depressive-like symptoms, such as the Porsolt swim test or sucrose preference tests. It is reasonable to assume that EE or other life style improvements may benefit families with a higher risk for disorders such as ADHD, schizophrenia, anxiety, and depression. The identification of miRNAs that mediate the actions of EE may facilitate the development of predictive biomarkers of disease and new therapeutic targets.

## Materials and Methods

### Experimental Design

The study involved forty-eight adult male Long-Evans hooded rats, bred and housed at the Canadian Centre for Behavioural Neuroscience. Rats were F3 offspring born to one of the following three maternal lineages: non-stress controls (n = 16), transgenerational prenatal stress exposure (*TPS*; n = 16), and multigenerational prenatal stress exposure (*MPS*; n = 16). TPS rats were the F3 generation of a filial line in which only the F0 dams were stressed during gestation. MPS rats were the F3 generation of a filial line in which dams from each consecutive generation (F0, F1, F2) were gestationally stressed. Each F3 group was gathered from 4 different litters (4 control, 4 TPS, 4 MPS for each group; n = 16 per group), and animals in each litter were then split into standard or enriched housing conditions (n = 8 pre group).

**Figure 6:**
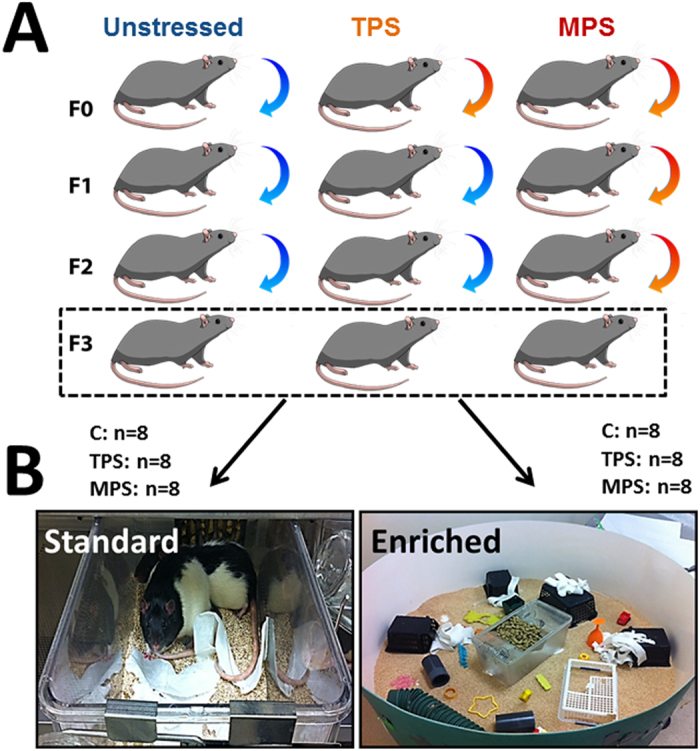
Animal model of transgenerational and multigenerational prenatal stress. The animal model included a non-stress control lineage, a transgenerational stress lineage in which gestational stress only occurred in F0 dams, and a multigenerational stress lineage in which F0-F1-F2 dams were stressed during pregnancy. Males from four F3 litters per group were tested. Four male rats per litter were used; 2 animals from each litter were placed in a standard environment and 2 rats from each litter were placed in an enriched environment at the age of 35 days.

At weaning, rats derived from the three lineages were assigned to either housing in standard cages, or housing in an enriched environment (EE). Thus, the following groups were tested: non-stress controls in standard (*Control*; n = 8) and EE (*Control-EE*; n = 8) housing conditions, TPS in standard (*TPS*; n = 8) and EE (*TPS-EE*; n = 8) housing, and MPS in standard (*MPS*; n = 8) and EE (*MPS-EE*; n = 8) housing. Figure 6 illustrates the experimental design of the present study.

The animals were housed under a 12 h light/dark cycle with lights on at 7:30 AM. The room temperature was maintained at 20 °C with relative humidity at 30%. Body weight was regularly recorded as the rats aged. Rats were assessed in the open field and elevated plus maze tasks at 35, 60–65 and 100 days old. At 100 days old, corticosterone (CORT) measurements were taken and at 180 days old, animals were euthanized for tissue collection. All procedures were performed in accordance with the guidelines of the Canadian Council on Animal Care and approved by the University of Lethbridge Animal Welfare Committee.

### Prenatal Stress

Pregnant dams were stressed by social isolation, which results in mild psychosocial stress^28^. The lack of social support was expected to alter physiology and subsequent behaviour^28^. For example, social isolation has been shown to have a profound effect on cognitive function and corticosterone levels in adult rats^73^. In this experiment, each dam was housed alone and did not experience any direct contact with conspecifics from P90 until the weaning of her offspring. Pairing for mating began at P90 and the maximum time spent in social isolation prior to conception was 30 days. Control rats were housed in pairs until gestational day 21.

### Standard and Enriched Housing Conditions

On P21, animals were weaned and assigned to two housing conditions. In the standard housing condition rats were housed in non-sibling pairs in Macrolon shoebox cages. From P21 to P35, the EE rats were housed in groups of four living in large Macrolon housing units. At P35, EE rats were moved to large circular housing units where they were housed in groups of eight. Enriched social interaction in the EE condition was particularly important given that animal lineages were exposed to social isolation stress. In previous studies, social interaction was shown to have a greater benefit than physical enrichment alone^74^. In addition to the increased social interactions and space, the EE was equipped with multiple shelters and toys that were exchanged weekly. In addition to standard rat chow, EE rats were regularly provided with novel types of foods, including raw pasta, non-sweetened breakfast cereal, and seeds. In total, three identically arranged EE were used in the study, each housing all of the rats from a single stress treatment group.

### Behavioural Analyses

#### Open Field Task

The open field task allows quantification of exploration of an aversive open arena^43^,^44^. The open field task was conducted using the VersaMax Legacy Open Field system (Omnitech Electronics, Inc., Dartmouth, NS, Canada; Fig. 4A), which measured an animal’s activity for a period of 10 minutes using an array of infrared sensors connected to a computer. Behavioural measures included total distance travelled *(Total Distance)* during the test interval (*Movement Time*), the amount of time spent within the margins of the open field (*Margin Time*), and time spent rearing (*Vertical Time*).

#### Elevated Plus Maze

The elevated plus maze (Fig. 4B) allows the assessment of anxiety-like behaviours in an aversive environment^75^. The elevated plus maze consisted of an opaque black Plexiglas maze suspended 50 cm above the ground. Two arms (50 cm × 10 cm) were enclosed by Plexiglas walls 40 cm high (“*closed arms*”) and two identically sized arms were without walls (“*open arms*”). Rats were placed in the center of the maze and allowed to explore freely for a period of five minutes. Behavioural measures included *total time* spent in the closed arms, open arms and at the end of open arms; *number of entries* into closed arms, open arms, and end of open arms; and *latency to enter* the closed arms as well as number of *risk assessment* behaviours (i.e. stretch-extend postures).

### Corticosterone Assay

Blood samples (0.6 ml) were collected from the lateral tail vein under 4% isoflurane anesthesia. Plasma CORT concentrations were determined by radioimmunoassay, ran in duplicates, using commercial kits (ELISA, Abcam Inc., ON, Canada).

### Tissue Collection

At the age of 180 days, 5 rats per group were euthanized with an overdose of Euthansol (Merck, QC, Canada) and perfused transcardially with PBS followed by 4% Paraformaldehyde (Sigma-Aldrich, MO, USA). Brains were extracted, stored in 4% PFA for 24 h and then transferred to sucrose solution for at least three days. Three rats per group were euthanized with a cardiac overdose of Euthansol and fresh prefrontal cortical tissue was extracted, frozen and processed for miRNA analysis.

### Histology

#### Immunohistochemistry

Brains were cut in coronal sections at a thickness of 40 μm and 12 series interval. Sections were processed for GR immunohistochemistry (mouse monoclonal antibody; Vector Labs) followed by biotinylated horse anti-mouse IgG (1:200; Vector Labs) and avidin-biotin-peroxidase complex (Vectastain Elite ABC Kits; Vector Labs).

#### Unbiased Stereology

All stereological data were blindly collected by a single investigator using Stereo Investigator (MicroBrightField Inc., 2013, Version 10). The density of glucocorticoid receptors (GRs) was calculated by dividing the cell numbers obtained with the optical fractionator by the volume of each interested region as calculated by Cavalieri’s principle^76^. Briefly, an optical fractionator probe was used to estimate the populations of hippocampal GRs, including areas of CA1/2, dentate gyrus (DG) and CA3. Systematically, randomly positioned grids (150 μm × 150 μm) containing counting frames (80 μm × 80 μm) were superimposed on the areas of investigation. A 120 μm × 120 μm grid was randomly placed on each section containing hippocampal areas. The Gundersen coefficient of error (CE) for cell number and volume estimates was ranging from 0.02–0.04 and 0.01–0.03, respectively^77^.

#### Neural Density Analysis – Cytoarchitectonics

Every third series of sections was mounted and stained with cresyl violet to detect Nissl bodies. The slides were captured using a motorized Zeiss AxioImager M1 microscope (Zeiss, Jena, Germany) at 1X magnification. The quantitative cytoarchitectonic analyses in cresyl violet-stained sections corresponding to an ROI measuring 0.766 mm^2^ slices at Bregma level 3.70 (caudal PFC) were performed with Image J V1.36 (http://rsb.info.nih.gov/ij/download.html). The “absolute grey level index” was ascertained as the measured parameter^78^. A step tablet was used (http://rsb.info.nih.gov/ij/download.html) to calibrate the optical density in the 8-bit images.

#### Prefrontal Cortical Thickness

PFC thickness was measured on cresyl stained sections at Bregma level 3.70 mm using ImageJ software (NIH). Thickness coordinates were compared to Paxinos and Watson rat atlas^79^ to determine location of measurement. The vertical (dorsoventral) distance was measured parallel to the midsagittal line, using the rhinal fissure endpoint to center the placement of the line. The horizontal (mediolateral) distance was measured perpendicular to the midsagittal line, crossing midway through the dorsoventral line^80^.

### MicroRNA Deep Sequencing

Deep sequencing of miRNA expression was performed using Illumina GAIIx genomic platform (Illumina, CA, USA). Briefly, base calling and demultiplexing was completed using CASAVA 1.8.1 software pipeline with default settings. Short read quality was examined using FastQC software. Adapters were trimmed using cutadapt software with options specified to search for adapters anywhere in the read sequence and retain only sequences over 15 nucleotides in length. Quality trimming was performed with Sanger quality score cutoff of 30. Over 92% were retained after trimming in each of the libraries. FastQC quality check was performed after trimming. MiRNA detection and counting was performed using standalone MicroRazerS 41 version 1.0^81^. Potential targets of selected miRNAs of interest were predicted using the 3′UTR available for Rat rn5 (UCSC) genome. Target prediction was based on miRanda v.3.3a (Computational Biology Center of Memorial Sloan-Kettering Cancer Center, NY, USA), an algorithm for finding genomic targets for microRNAs, with default options. Gene set enrichment analysis was performed using GOstats package v.2.34 (Bioconductor, MD, USA) as a way to identify significantly over-represented gene ontology categories and KEGG pathways^82^,^83^.

### Statistical Analysis

Statistical computations were based on Statview software version 5.0 (SAS Institute, NC, USA). All behavioural measures were analyzed on a per-variable basis using a mixed-design analysis of variance (ANOVA). The between-group independent variables included stress treatment (non-stress controls, TPS, MPS) and housing (standard and EE). The within-group variable was time. Post-hoc comparisons were performed using Tukey HSD for between-enrichment group differences in groups, and independent sample t-tests for between-group differences for enrichment. Additionally, non-parametric correlation was conducted to determine the relationship between the dependent variables. A *p*-value of less than 0.05 was chosen as the significance level.

For miRNA analysis, raw count data underwent normalization and regularized *log* transformation using statistical routines implemented in the DESeq2 bioconductor package^84^ as described in the DESeq2 user manual. Pairwise comparisons between experimental groups were performed using DESeq2 with default settings applied to normalization and statistical testing. Small RNAs with false discovery rate adjusted p-values <0.1 were considered differentially expressed.

## Additional Information

**How to cite this article**: McCreary, J. K. *et al*. Environmental Intervention as a Therapy for Adverse Programming by Ancestral Stress. *Sci. Rep.* **6**, 37814; doi: 10.1038/srep37814 (2016).

**Publisher's note:** Springer Nature remains neutral with regard to jurisdictional claims in published maps and institutional affiliations.

## Supplementary Material

Supplementary Information
