# Supplementary material for: Environmental Intervention as a Therapy for Adverse Programming by Ancestral Stress
Source: Sci Rep. 2016 Nov 24;6:37814. doi: 10.1038/srep37814 (PMC5121646; doi:10.1038/srep37814)
Supplement: Supplementary Information [file srep37814-s1.doc]

**SUPPLEMENTAL MATERIAL**

**Environmental Intervention as a Therapy for Adverse Programming by Ancestral Stress**

*J. Keiko McCreary^1^, Zachary T. Erickson^1^, YongXin Hao^1^, Yaroslav Ilyntskyy^2^, Igor Kovalchuk^2^, Gerlinde A.S. Metz^1^*

Supplemental Figures: 2

Supplemental Tables: 1


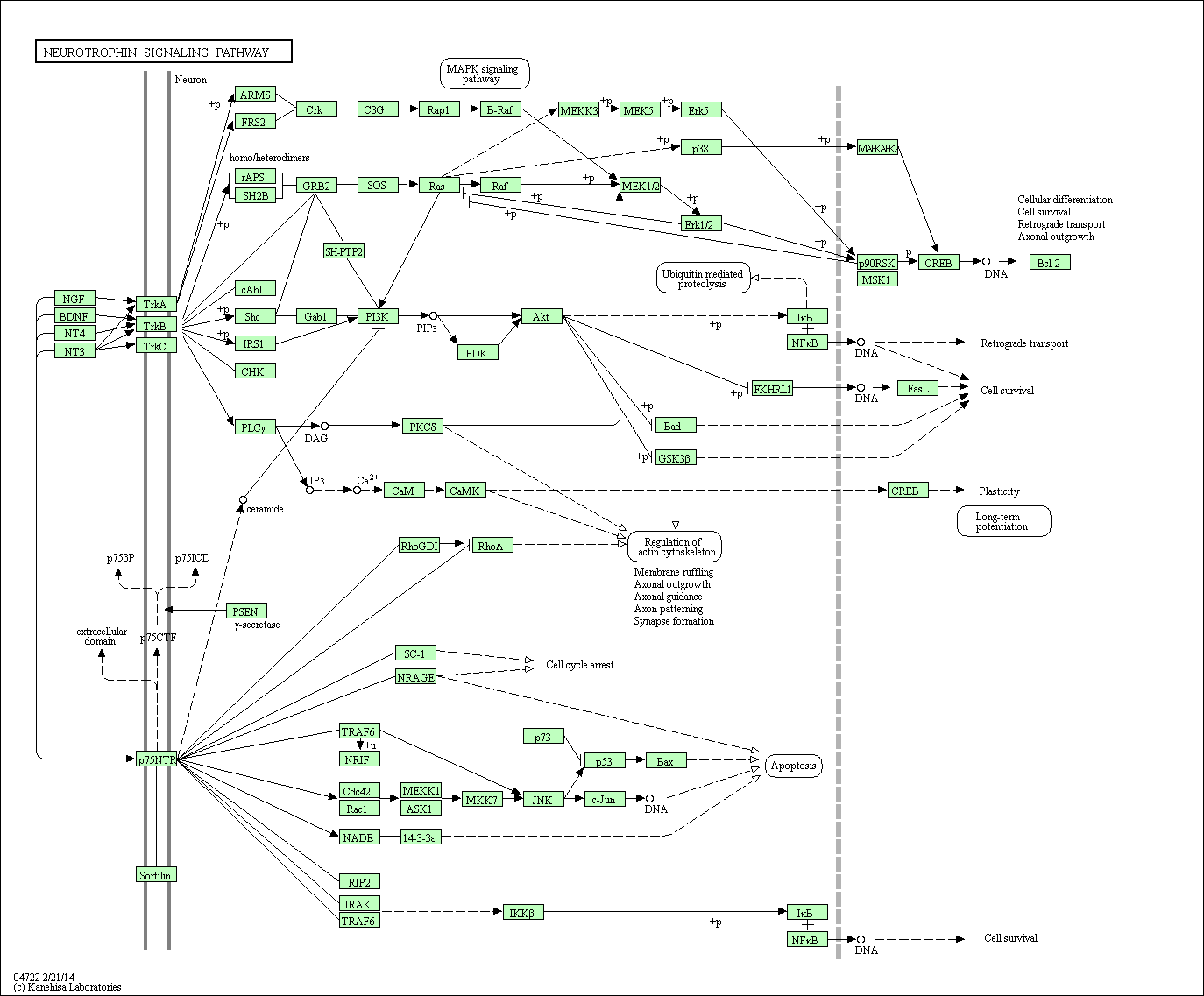


**FIGURE S1: Neurotrophin signaling pathway as target for environmentally-regulated miR-182 expression.** Specifically, the 3'UTRs of BDNF and NT-3 genes show up as high scoring targets in miR-182 binding prediction. The pathway suggests a plausible mechanistic link between miRNA-mediated post-transcriptional regulation of BDNF and NT-3 and EE-associated behavioural and endocrine improvements.

**
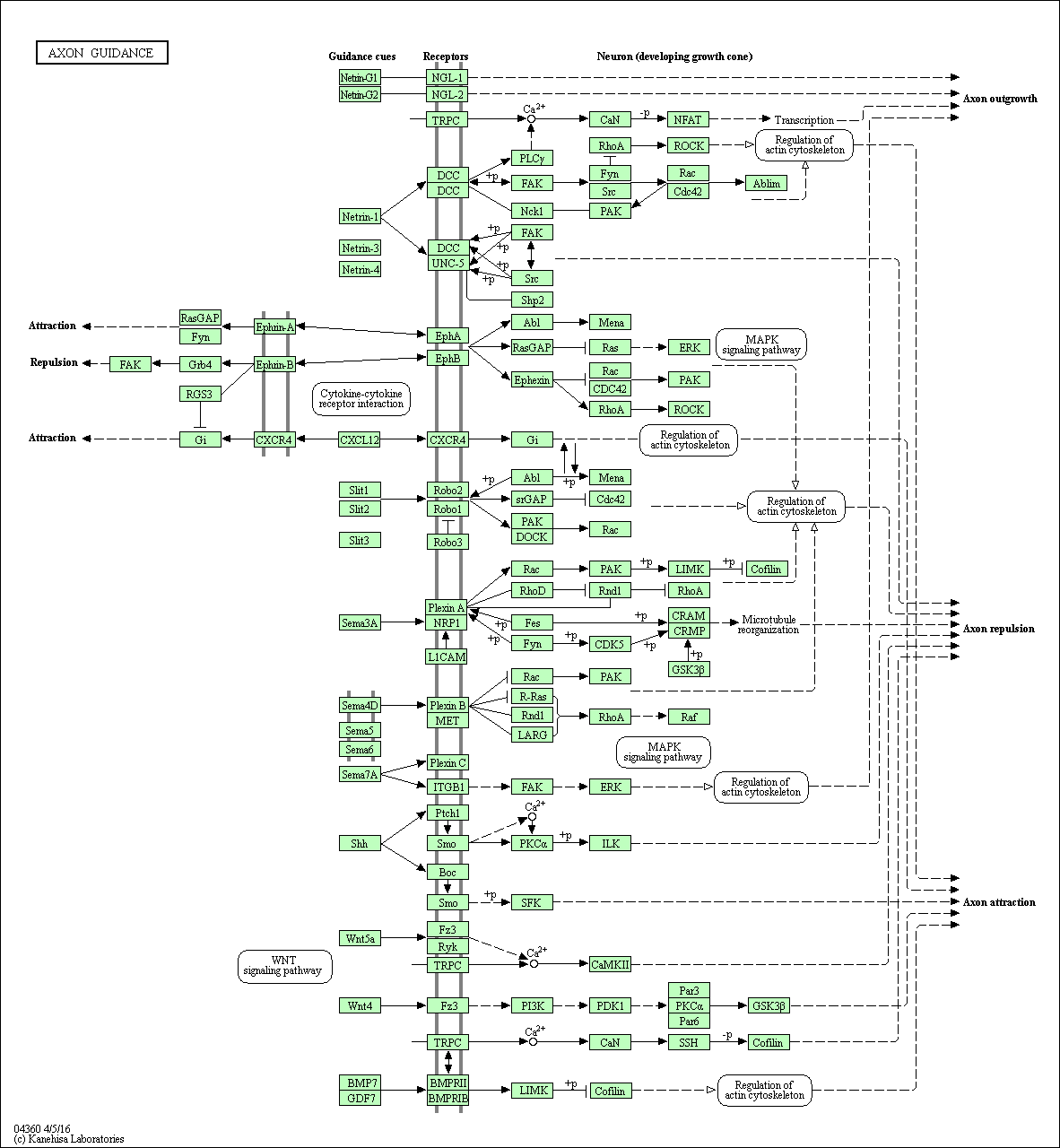
**

**FIGURE S2: Axon guidance signaling pathway as target for environmentally-regulated miR-182 expression.** Specific potential targets include netrin (G1), semaphorin (Sema3A, 5, 6), ephrin (A), Slit1 and Slit2, and their receptors. The 3'UTRs of these genes show up as high scoring targets in miR-182 binding prediction. The pathway suggests that miR-182 may regulate axonal pathfinding during brain development and maturation, which may be altered by transgenerational stress and housing conditions.

**TABLE S1.** Small RNA expression fold change with raw and adjusted p-value. Included are values with a p-value<0.1. C, control; MPS, multigenerational prenatal stress; TPS, transgenerational prenatal stress; EE, Enriched Environment housing condition. In the order of smallest adjusted p-values. (NA indicates p-adj >1).

| **Treatment** | **small RNA** | **log2FoldChange** | **SE** | **Wald Statistic** | **p-value** | **p-adj.** |
| --- | --- | --- | --- | --- | --- | --- |
| C-EE vs C | rno-miR-582-5p | 1.01799 | 0.50192 | 2.02819 | 0.04254 | 0.99394 |
| C-EE vs C | rno-miR-29c-5p | -1.08965 | 0.56240 | -1.93748 | 0.05268 | 0.99394 |
| C-EE vs C | FR0192132|Piwi-interacting | 1.049282 | 0.59089 | 1.77574 | 0.07577 | 0.99394 |
| C-EE vs C | rno-miR-1306-5p | 1.045943 | 0.59091 | 1.77004 | 0.07671 | 0.99394 |
| C-EE vs C | rno-miR-125a-3p | 1.033198 | 0.58526 | 1.76535 | 0.07750 | 0.99394 |
| C-EE vs C | FR0313594|C/D | -1.05611 | 0.62807 | -1.68150 | 0.09266 | 0.99394 |
| C-EE vs C | rno-miR-1843b-5p | -0.33416 | 0.20298 | -1.64621 | 0.09971 | 0.99394 |
| MPS vs C | rno-miR-124-3p | 2.938383 | 0.19486 | -3.28492 | 0.00100 | 0.08160 |
| MPS vs C | rno-miR-182 | 1.55158 | 0.49857 | -2.76247 | 0.00573 | 0.30594 |
| MPS vs C | rno-miR-486 | 2.758066 | 0.18923 | -2.03388 | 0.04196 | 0.98750 |
| MPS vs C | rno-miR-10a-5p | 4.505499 | 0.59672 | -2.02393 | 0.04297 | 0.98750 |
| MPS vs C | rno-miR-24-3p | 1.108923 | 0.21318 | -1.98813 | 0.04679 | 0.98750 |
| MPS vs C | rno-let-7b-3p | 2.90867 | 0.29584 | 1.96805 | 0.04906 | 0.98750 |
| MPS vs C | rno-miR-411-5p | 0.746015 | 0.20241 | 1.96533 | 0.04937 | 0.98750 |
| MPS vs C | rno-miR-103-3p | 0.313884 | 0.17247 | 1.81985 | 0.06878 | 0.99720 |
| MPS vs C | rno-miR-132-3p | 0.280005 | 0.15908 | 1.76011 | 0.07838 | 0.99720 |
| MPS vs C | rno-miR-873-3p | -0.60339 | 0.35892 | -1.68111 | 0.09274 | 0.99720 |
| MPS vs C | rno-miR-541-5p | 0.466462 | 0.28692 | 1.62571 | 0.10401 | 0.99720 |
| MPS vs C | rno-miR-191a-5p | 0.371252 | 0.23036 | 1.61156 | 0.10705 | 0.99720 |
| MPS vs C | rno-miR-434-5p | -0.78145 | 0.48660 | -1.60590 | 0.10829 | 0.99720 |
| MPS vs C | rno-miR-27a-3p | -0.43036 | 0.26869 | -1.60169 | 0.10922 | 0.99720 |
| MPS vs C | Rattus_norvegicus_chr2.trna4358-GlyCCC | 2.935006 | 0.38612 | 7.60117 | 2.93E-14 | 4.70E-12 |
| MPS vs C | Rattus_norvegicus_chr1.trna2048-SeC(e)TCA | 1.765595 | 0.61820 | 2.85598 | 0.00429 | NA |
| MPS vs C | FR0024423|Piwi-interacting | 2.16298 | 0.76316 | 2.83423 | 0.00459 | NA |
| MPS vs C | FR0403295|C/D | 2.070462 | 0.76411 | 2.70962 | 0.00673 | NA |
| MPS vs C | rno-miR-3553 | 1.289105 | 0.68387 | -2.48191 | 0.01306 | NA |
| MPS vs C | rno-miR-3577 | 1.728112 | 0.74481 | 2.32018 | 0.02033 | NA |
| MPS vs C | rno-miR-342-5p | -1.59533 | 0.76788 | -2.07757 | 0.03774 | NA |
| MPS vs C | FR0392512|H/ACA | 1.485154 | 0.75781 | 1.95977 | 0.05002 | NA |
| MPS vs C | FR0249489|Piwi-interacting | 1.200728 | 0.63571 | 1.88879 | 0.05891 | NA |
| MPS vs C | FR0327571|Piwi-interacting | 1.391373 | 0.76610 | 1.81616 | 0.06934 | NA |
| MPS vs C | ENSRNOG00000040834|5S | -1.25947 | 0.72337 | -1.74119 | 0.08166 | NA |
| MPS vs C | rno-miR-30d-3p | 0.786133 | 0.45271 | 1.73647 | 0.08248 | NA |
| MPS vs C | FR0107401|Piwi-interacting | 0.998947 | 0.60326 | 1.65590 | 0.09774 | NA |
| MPS vs C | FR0234140|C/D | -1.23158 | 0.74590 | -1.65112 | 0.09871 | NA |
| MPS vs C | rno-miR-1306-5p | 1.145874 | 0.70816 | 1.61810 | 0.10564 | NA |
| MPS vs MPS-EE | rno-miR-199a-3p | 1.40123 | 0.43517 | 3.21993 | 0.00128 | 0.96806 |
| MPS vs MPS-EE | rno-miR-204-5p | 1.161981 | 0.41687 | 2.78736 | 0.00531 | 0.99069 |
| MPS vs MPS-EE | rno-miR-219a-5p | 1.462179 | 0.61631 | 2.37245 | 0.01767 | 0.99069 |
| MPS vs MPS-EE | rno-miR-375-3p | 1.072906 | 0.45396 | 2.36339 | 0.01810 | 0.99069 |
| MPS vs MPS-EE | rno-miR-21-5p | 0.912857 | 0.38922 | 2.34534 | 0.01900 | 0.99069 |
| MPS vs MPS-EE | rno-miR-211-5p | 1.437792 | 0.62700 | 2.29312 | 0.02184 | 0.99069 |
| MPS vs MPS-EE | rno-miR-7a-5p | 0.956106 | 0.45648 | 2.09443 | 0.03622 | 0.99069 |
| MPS vs MPS-EE | FR0024423|Piwi-interacting | -1.28556 | 0.66814 | -1.92409 | 0.05434 | 0.99069 |
| MPS vs MPS-EE | rno-miR-3544 | 1.183926 | 0.62827 | 1.88439 | 0.05951 | 0.99069 |
| MPS vs MPS-EE | rno-miR-26b-5p | 0.626912 | 0.34044 | 1.84143 | 0.06555 | 0.99069 |
| MPS vs MPS-EE | rno-miR-450a-5p | 0.910405 | 0.49587 | 1.83595 | 0.06636 | 0.99069 |
| MPS vs MPS-EE | FR0077443|Piwi-interacting | 0.901941 | 0.50513 | 1.78554 | 0.07417 | 0.99069 |
| MPS vs MPS-EE | rno-miR-200c-3p | 1.091507 | 0.61228 | 1.78268 | 0.07463 | 0.99069 |
| MPS vs MPS-EE | FR0059851|Piwi-interacting | 1.029639 | 0.59966 | 1.71701 | 0.08597 | 0.99069 |
| MPS vs MPS-EE | rno-miR-106b-5p | 0.936773 | 0.55127 | 1.69927 | 0.08926 | 0.99069 |
| MPS vs MPS-EE | rno-let-7d-3p | 0.473828 | 0.27892 | 1.69879 | 0.08935 | 0.99069 |
| MPS vs MPS-EE | rno-miR-145-5p | 0.552868 | 0.32931 | 1.67884 | 0.09318 | 0.99069 |
| MPS vs MPS-EE | rno-let-7e-3p | 0.988459 | 0.59301 | 1.66684 | 0.09554 | 0.99069 |
| MPS vs MPS-EE | rno-miR-136-3p | -0.33601 | 0.20291 | -1.65598 | 0.09773 | 0.99069 |
| MPS vs MPS-EE | rno-miR-200b-5p | 0.987452 | 0.59807 | 1.65104 | 0.09873 | 0.99069 |
| MPS vs MPS-EE | rno-miR-187-3p | 1.019519 | 0.62172 | 1.63981 | 0.10104 | 0.99069 |
| MPS vs MPS-EE | rno-miR-152-3p | 1.076068 | 0.66893 | 1.60861 | 0.10770 | 0.99069 |
| TPS vs C | FR0403295|C/D | 3.474859 | 0.81971 | 4.23910 | 2.24E-05 | 0.01210 |
| TPS vs C | rno-miR-10a-5p | 7.276458 | 0.61994 | -4.15206 | 3.29E-05 | 0.01210 |
| TPS vs C | Rattus_norvegicus_chr2.trna4358-GlyCCC | 1.638738 | 0.40991 | 3.99778 | 6.39E-05 | 0.01566 |
| TPS vs C | rno-miR-182 | 1.499731 | 0.48585 | -3.47907 | 0.00050 | 0.02453 |
| TPS vs C | rno-miR-124-3p | 3.576407 | 0.18801 | -3.10760 | 0.00188 | 0.02772 |
| TPS vs C | rno-miR-3553 | 1.351694 | 0.77179 | -2.71746 | 0.00657 | 0.80584 |
| TPS vs C | rno-miR-486 | 8.211783 | 0.16830 | -2.62079 | 0.00877 | 0.92111 |
| TPS vs C | rno-miR-429 | -1.82056 | 0.80683 | -2.25648 | 0.02404 | 0.99689 |
| TPS vs C | rno-miR-3577 | 1.746913 | 0.78483 | 2.22582 | 0.02602 | 0.99689 |
| TPS vs C | rno-miR-3102 | 1.222738 | 0.55617 | 2.19846 | 0.02791 | 0.99689 |
| TPS vs C | ENSRNOG00000034753|5S | -1.86927 | 0.85373 | -2.18950 | 0.02856 | 0.99689 |
| TPS vs C | ENSRNOG00000034421|5S | -1.80827 | 0.83891 | -2.15549 | 0.03112 | 0.99689 |
| TPS vs C | rno-miR-30b-3p | 1.761104 | 0.81981 | 2.14818 | 0.03169 | 0.99689 |
| TPS vs C | ENSRNOG00000034612|5S | -1.68545 | 0.83485 | -2.01885 | 0.04350 | 0.99689 |
| TPS vs C | rno-miR-667-3p | 0.82052 | 0.40800 | 2.01103 | 0.04432 | 0.99689 |
| TPS vs C | ENSRNOG00000034413|5S | -1.7104 | 0.85375 | -2.00338 | 0.04513 | 0.99689 |
| TPS vs C | ENSRNOG00000041881|5S | -1.67308 | 0.84934 | -1.96985 | 0.04885 | 0.99689 |
| TPS vs C | rno-miR-128-2-5p | 1.645695 | 0.84239 | 1.95360 | 0.05074 | 0.99689 |
| TPS vs C | rno-miR-135b-5p | -0.90651 | 0.46417 | -1.95295 | 0.05082 | 0.99689 |
| TPS vs C | ENSRNOG00000041036|5S | -1.5774 | 0.82762 | -1.90594 | 0.05665 | 0.99689 |
| TPS vs C | ENSRNOG00000035238|5S | -1.54693 | 0.84730 | -1.82569 | 0.06789 | 0.99689 |
| TPS vs C | rno-miR-30d-3p | 0.754134 | 0.42095 | 1.79150 | 0.07321 | 0.99689 |
| TPS vs C | rno-miR-541-5p | 0.565428 | 0.31675 | 1.78505 | 0.07425 | 0.99689 |
| TPS vs C | rno-miR-151-3p | 0.442087 | 0.25216 | 1.75317 | 0.07957 | 0.99689 |
| TPS vs C | rno-miR-25-5p | 1.420034 | 0.82786 | 1.71529 | 0.08629 | 0.99689 |
| TPS vs C | rno-miR-141-3p | -1.24529 | 0.74211 | -1.67803 | 0.09334 | 0.99689 |
| TPS vs C | rno-miR-671 | 0.776569 | 0.46780 | 1.66002 | 0.09690 | 0.99689 |
| TPS vs C | FR0062876|Piwi-interacting | -1.36453 | 0.82368 | -1.65660 | 0.09760 | 0.99689 |
| TPS vs C | ENSRNOG00000041010|5S | -1.36884 | 0.84180 | -1.62607 | 0.10393 | 0.99689 |
| TPS vs C | ENSRNOG00000040834|5S | -1.37447 | 0.84555 | -1.62552 | 0.10405 | 0.99689 |
| TPS vs TPS-EE | rno-miR-29c-5p | -1.08965 | 0.56240 | -1.93748 | 0.05268 | 0.99341 |
| TPS vs TPS-EE | rno-miR-582-5p | 1.01799 | 0.50192 | 2.02819 | 0.04254 | 0.99394 |
| TPS vs TPS-EE | FR0192132|Piwi-interacting | 1.049282 | 0.59089 | 1.77574 | 0.07577 | 0.99394 |
| TPS vs TPS-EE | rno-miR-1306-5p | 1.045943 | 0.59092 | 1.77004 | 0.07671 | 0.99394 |
| TPS vs TPS-EE | rno-miR-125a-3p | 1.033198 | 0.58526 | 1.76535 | 0.07750 | 0.99394 |
| TPS vs TPS-EE | FR0313594|C/D | -1.05611 | 0.62807 | -1.68150 | 0.09266 | 0.99394 |
| TPS vs TPS-EE | rno-miR-1843b-5p | -0.33416 | 0.20298 | -1.64621 | 0.09971 | 0.99394 |
